# Robust Weighted Sum Harvested Energy Maximization for SWIPT Cognitive Radio Networks Based on Particle Swarm Optimization

**DOI:** 10.3390/s17102275

**Published:** 2017-10-06

**Authors:** Pham Viet Tuan, Insoo Koo

**Affiliations:** 1 School of Electrical and Computer Engineering, University of Ulsan, Ulsan 680-749, Korea; phamviettuan@gmail.com; 2 Faculty of Physics, University of Education, Hue University, 34 Le Loi Str., Hue City 530000, Vietnam

**Keywords:** cognitive radio networks (CRNs), simultaneous wireless information and power transfer (SWIPT), power-splitting, semidefinite relaxation (SDR), particle swarm optimization (PSO)

## Abstract

In this paper, we consider multiuser simultaneous wireless information and power transfer (SWIPT) for cognitive radio systems where a secondary transmitter (ST) with an antenna array provides information and energy to multiple single-antenna secondary receivers (SRs) equipped with a power splitting (PS) receiving scheme when multiple primary users (PUs) exist. The main objective of the paper is to maximize weighted sum harvested energy for SRs while satisfying their minimum required signal-to-interference-plus-noise ratio (SINR), the limited transmission power at the ST, and the interference threshold of each PU. For the perfect channel state information (CSI), the optimal beamforming vectors and PS ratios are achieved by the proposed PSO-SDR in which semidefinite relaxation (SDR) and particle swarm optimization (PSO) methods are jointly combined. We prove that SDR always has a rank-1 solution, and is indeed tight. For the imperfect CSI with bounded channel vector errors, the upper bound of weighted sum harvested energy (WSHE) is also obtained through the S-Procedure. Finally, simulation results demonstrate that the proposed PSO-SDR has fast convergence and better performance as compared to the other baseline schemes.

## 1. Introduction

Radio frequency energy harvesting is emerging as an active research area in the fields of both academics and industry, due to the fact that it can solve the bottle-neck of battery-powered wireless devices. It is especially important and useful in wireless sensor networks since sensor nodes have a limited amount of energy. In this new paradigm, the RF can bring both data and power from transmitters to receivers [1,2,3,4,5,6,7,8], referred to as “simultaneous wireless information and power transfer (SWIPT)”. Thus, new pre-coding techniques for transmitters and optimizing design techniques for receivers need to be investigated. The receiver operates in two modes where it switches between information decoding (ID) and energy harvesting (EH), i.e., time-switching (TS) mode, or shares the incoming signal into EH and ID parts, i.e., power-splitting (PS) [1]. Joint power-splitting SWIPT and beamforming are applied to some conventional networks such as multiuser multi-input single-output (MISO) systems [3,4] where transmission energy and energy efficiency are optimized, respectively. These research issues are also considered in systems with multi-antenna transmitters and receivers [5], cooperative networks [6], and interference channels [7], as well as robust secure transmission [8]. In addition, the authors in [9,10,11] studied the transmit power minimization problem in multiuser SWIPT MISO systems under the imperfect channel side information (CSI) with bounded channel vector errors and stochastic channel vector errors. As an important criterion, the sum harvested energy maximization in multiuser power-splitting SWIPT MISO system is investigated under the perfect channel side information of channels from transmitters to receivers [12]. However, the solution of [12] based on successive second-order cone programming cannot be applied to cognitive radio networks where interference threshold constraints of primary users should be considered with the case of imperfect CSI.

Similar to the works introduced by Goldsmith et al. [13], the applications of RF-powered techniques to harvest energy and transfer data in cognitive radio networks (CRNs) were summarized by Mohjazi et al. [14]. Other researchers studied SWIPT for different scenarios in order to provide energy to receivers while ensuring quality of service. In [15], the secondary network exploited both spectrum and energy in primary networks while assisting primary data transmission. In [16], Ng et al. studied one secondary data link in the presence of multiple energy harvesting receivers and primary users. Yang et al. in [17] and Lee et al. in [18] also investigated SWIPT in cognitive relay and cognitive wirelessly powered networks, respectively. However, CRNs with power-splitting SWIPT and beamforming designs in multiuser scenarios have not been well studied so far. The studies on power-splitting SWIPT in CRNs have many potential applications, such as wirelessly powered cognitive sensor networks and cognitive cellular networks, where users need to both receive information and obtain energy. In the our prior work [19], we considered the SWIPT cognitive radio network scenarios, and designed the system by considering the following important two criteria: one is “max–min harvested energy of cognitive users” and the other is “the worst-user trade-off between harvested energy of cognitive users and interference power of primary users” under the perfect CSI. Another important criterion in SWIPT cognitive radio networks is “weighted sum harvested energy maximization” under both perfect CSI and imperfect CSI cases, which have not been fully investigated yet.

To the best of our knowledge, this is the first work that investigates a multiuser power-splitting SWIPT for CRN in which one secondary transmitter (ST) equipped with a multi-antenna will transmit information and energy to multiple single-antenna secondary receivers (SRs) that have a PS structure, in the existence of multiple single-antenna primary users (PUs). The primary goal of this paper is to maximize the weighted sum harvested energy (WSHE) of all SRs by jointly optimizing the transmit beamforming vectors and the PS ratios while satisfying the minimum requirement of each SR’s signal-to-interference-plus-noise ratio (SINR), the ST’s limited transmission power, and each PU’s specified interference. It is noteworthy that the harvested energy received by an SR can be controlled by adjusting the weighted factors at the ST. The user-centric energy criteria such as max–min fairness harvested energy or the trade-off between EH and interference are studied in our prior work [19] where the more energy is transmitted over the poor channel to combat the channel attenuation for the worst user. Therefore, the worst user is always guaranteed in both information and energy. Unlike [19], in this paper the system-centric WSHE criterion is investigated where the total harvested energy of system is maximized regardless of the harvested energy of worst user. Thus, more energy is transmitted over the better channel, while the information rate is still guaranteed for every cognitive user. In addition, in [19], we only studied the problems for the ideal case with the perfect CSI. Unlike [19], we in this paper also study the WSHE maximization problem in the practical scenario under the imperfect CSI with errors bound. It is noted that this realistic case frequently occurs in wireless cognitive sensor networks with low complexity sensor nodes.

More specifically, in this paper we solve two completely novel research issues that in prior works have not been studied. The first one is WSHE maximization in multi-user SWIPT cognitive radio networks under the perfect CSI. The other issue is consideration of the WSHE problem under the imperfect CSI. The main contributions of this paper can be summarized as follows.

- For the perfect CSI, we formulate the non-convex optimization problem for the WSHE of all secondary receivers including constraints to limited transmission power at the ST, minimum required SINR at each SR, and interference threshold at each PU. The objective function is non-convex due to the coupled design variables of both the transmit beamforming vectors at the ST and the PS ratios at the SRs. Therefore, we solve it in two steps based on two variable groups of beamforming vectors and PS ratios. In the first step, we fix the PS ratios and obtain the optimal beamforming vectors by applying a semidefinite relaxation (SDR) technique. Interestingly, we can show that SDR is tight for our problem. In the second step, we propose an algorithm based on particle swarm optimization (PSO) to find the approximate optimal PS ratios. The baseline schemes are considered for performance comparison, in which the zero-forcing beamforming vectors at the ST and the equal power splitting ratios at the SRs are applied.
- For the imperfect CSI, the S-Procedure and SDR technique are applied to recast the robust WSHE maximization problem as a semidefinite programming (SDP) problem. Then, the similar PSO-based method is used to solve the formulated problem. However, we only obtain the upper bound value of WSHE since the optimal solutions are unlikely to satisfy the rank-1 constraints.
- Finally, simulation results show that the proposed PSO-SDR has fast convergence and better performance compared to the other baseline schemes. In addition, the the proposed PSO-SDR converges to the optimal value, achieved by the brute-force search (BFS) method, while obtaining significantly lower computational complexity.

The remainder of the paper is organized as follows. The system description is presented in Section 2. The weighted sum harvested energy maximization problem in the perfect CSI case is solved in Section 3 by the joint SDR technique and PSO method. Section 4 presents the solution of the robust WSHE maximization problem in the imperfect CSI case. The simulation results are provided in Section 5, followed by conclusions in Section 6.

**Notation** **1.** Vectors and matrices are indicated by boldface lower case and capital letters, respectively. $X^{*}$ and $X^{H}$ represent the conjugate and Hermitian transpose of matrix X, respectively. We use $\mathrm{Tr}\left(X\right)$, and $\mathrm{rank}\left(X\right)$ to indicate trace and rank of matrix X, respectively. X⪰0 and X≻0 indicate that matrix X is positive semidefinite or positive definite, respectively. The Euclidean norm of a complex vector and the absolute value of a complex scalar are represented by $∥\;\cdot \;∥$ and $\left|\;\cdot \;\right|$, respectively. The space of m×n matrices with complex entries is denoted by $C^{m\times n}$ . $H^{N}$ denotes the space of N×N Hermitian matrices. I denotes the identity matrix with appropriate size. $\;CN\left(\mu ,\sigma^{2}\right)$ represents the distribution of a circularly symmetric complex Gaussian (CSCG) random variable with mean μ and variance $\sigma^{2}$, and ’∼’ means “distributed as". Moreover, we summarize the important abbreviations and explain their meanings in Table 1.

## 2. System Description

**Figure 1:**
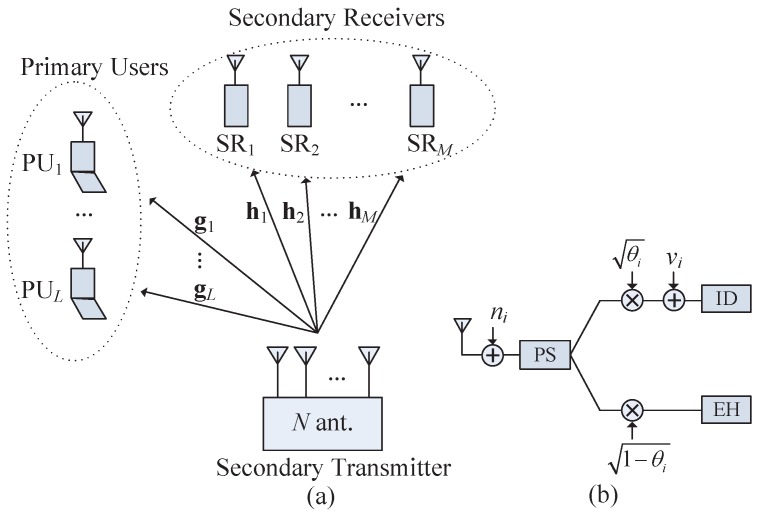
(**a**) The multi-input single-output (MISO) cognitive radio network being considered in the paper, and (**b**) the power-splitting receive model at ${\mathrm{SR}}_{i}$. PU: primary user; SR: secondary receiver; PS: power splitting; ID: information decoding; EH: energy harvesting.

Figure 1a shows a considered MISO cognitive radio networks with SWIPT capability that includes one ST, *M* SRs, and *L* PUs. *M* information messages are simultaneously sent by the ST equipped with *N* antennas to *M* SRs equipped with a single antenna at the same frequency as the PU’s communication when *L* single-antenna PUs exist. We denote $h_{i}\in C^{N\times 1}$, $i\in \left\{1,...,M\right\}$ and $g_{l}\in C^{N\times 1}$, $l\in \left\{1,...,L\right\}$ as the baseband equivalent channels from the ST to $SR_{i}$, and $PU_{l}$, respectively. The ST is supposed to obtain these channel vectors perfectly with their elements as independent and identically distributed (i.i.d) circularly symmetric complex Gaussian (CSCG) variables.

The secondary transmitter can practically achieve channel side information (CSI) vectors from the ST to the SRs by utilizing the conventional channel estimation methods. First, the pilots are transmitted by the ST, and then the ST obtains feedback on the results of channel estimation from the SRs. In addition, when there is cooperation between licensed and cognitive systems, the channel estimation can be sent from the PUs directly to the ST via the pilot signals from the cognitive network or indirectly through a frequency manager [23]. In an other way, the ST can achieve link information by periodically detecting the signal sent by the PUs since the licensed network utilizes time division duplex (TDD) for communication. However, it is difficult for the ST to obtain perfect instantaneous CSI for both cross and direct links. Thus, we also consider the harvested energy of the SRs for imperfect CSI case in the paper.

The ST sends the communication signal, which can be represented as follows

(1) $$x\;=\;\sum_{i=1}^{M}w_{i}s_{i},$$

where $s_{i}\in C$ is the symbol which carries information addressed to $SR_{i}$ with $E\left\{{\left|s_{i}\right|}^{2}\right\}=1$, and the corresponding the precoding beamforming vector $w_{i}\in C^{N\times 1}$. The average transmission power is calculated as $P=\sum_{i=1}^{M}{∥w_{i}∥}^{2}$. The over-the-air transmit power is limited by the maximum transmit power $P_{\max}$ [24,25]. Although the power amplifier efficiency and the constant circuit power consumption of the secondary transmitter accounting for antenna circuits, transmit filter, mixer, frequency synthesizer, and digital-to-analog converter, etc., [24,25] are important parameters in considering energy system consumption, these factors will not affect our main objective maximizing the harvested energy at secondary users under the limited transmit power. Therefore, we neglect these parameters when evaluating the sum harvested energy problem. The baseband signal received at $SR_{i}$ is represented as follows

(2) $$r_{i}\;=\;h_{i}^{H}w_{i}s_{i}+h_{i}^{H}\sum_{j=1,j\neq i}^{M}w_{j}s_{j}+\;n_{i},\;\forall i,$$

where $n_{i}∼\;CN\left(0,\sigma_{n}^{2}\right)$ is is the antenna noise added at $SR_{i}$.

In the system, the SRs equipped with the PS receive structure shown in Figure 1b can simultaneously decode information and harvest energy from incoming signals. Under this PS scheme, $SR_{i}$ splits the incoming signal into two streams, where one stream with power ratio $\theta_{i}\in \left(0,1\right)$ is utilized for ID, and the other with power ratio $\left(1-\theta_{i}\right)$ is utilized for EH. Figure 1b shows that the incoming signal for ID is $r_{i,\mathrm{ID}}\;=\;\sqrt{\theta_{i}}\;r_{i}+\;v_{i}$ where $v_{i}∼\;CN\left(0,\sigma_{v}^{2}\right)$ is the circuit noise appended by the ID of $SR_{i}$. Thus, the SINR at $SR_{i}$ is calculated as

(3) $${\mathrm{SINR}}_{i}\;=\;\frac{\theta_{i}{\left|h_{i}^{H}w_{i}\right|}^{2}}{\theta_{i}\left(\sum_{j=1,j\neq i}^{M}{\left|h_{i}^{H}w_{j}\right|}^{2}\;+\;\sigma_{n}^{2}\right)+\sigma_{v}^{2}}\;,\;\forall i.$$

Moreover, the incoming signal for EH of $SR_{i}$ is $r_{i,\mathrm{EH}}\;=\;\sqrt{1-\theta_{i}}\;r_{i}$. The initial energy can exist at the beginning of each time block and we assume that the size of battery is large enough to store both initial energy and harvested energy. We here focus on jointly optimizing precoding beamforming vectors at the transmitter and power splitting ratios at the secondary receivers so that the SRs can harvest maximum energy.

Then, the energy harvested by the EH of $SR_{i}$ is computed as follows

(4) $$EH_{i}=\eta_{i}\left(1-\theta_{i}\right)\left(\sum_{j=1}^{M}{\left|h_{i}^{H}w_{j}\right|}^{2}\;+\;\sigma_{n}^{2}\right),\;\;\forall i,$$

where the efficiency of $SR_{i}$’s energy harvester is denoted by $\eta_{i}\in \left(0,1\right]$. Furthermore, the power of interference at $PU_{l}$ caused by the ST is computed as

(5) $$IT_{l}=\sum_{i=1}^{M}{\left|g_{l}^{H}w_{i}\right|}^{2},\;\;\forall l.$$

## 3. Problem Formulation and Solution

In this paper, the main objective is to maximize the WSHE of all SRs by jointly finding the received PS ratios $\left\{\theta_{i}\right\}$ and the beamforming vectors $\left\{w_{i}\right\}$ subject to the required SINR at each SR, the maximum transmit power at the ST, and the interference threshold for each PU. Therefore, the WSHE problem can be expressed as follows

(6a) $$\begin{array}{cc} \max_{\left\{w_{i}\right\},\left\{\theta_{i}\right\}\;}\;\; & \sum_{i=1}^{M}\lambda_{i}\eta_{i}\left(1-\theta_{i}\right)\left(\sum_{j=1}^{M}{\left|h_{i}^{H}w_{j}\right|}^{2}\;+\;\sigma_{n}^{2}\right) \end{array}$$

(6b) $$\begin{array}{cc} s.t.\;\;\;\; & \sum_{i=1}^{M}{∥w_{i}∥}^{2}\leq \;P_{\max} \end{array}$$

(6c) $$\begin{array}{c} \frac{\theta_{i}{\left|h_{i}^{H}w_{i}\right|}^{2}}{\theta_{i}\left(\sum_{j=1,j\neq i}^{M}{\left|h_{i}^{H}w_{j}\right|}^{2}\;+\;\sigma_{n}^{2}\right)+\sigma_{v}^{2}}\;\geq \;a_{i}\;,\;\forall i,\;\forall i \end{array}$$

(6d) $$\begin{array}{c} \sum_{i=1}^{M}{\left|g_{l}^{H}w_{i}\right|}^{2}\leq \;\;I_{t},\;\forall l \end{array}$$

(6e) $$\begin{array}{c} 0<\theta_{i}<1,\;\forall i, \end{array}$$

where we set $\sum_{i=1}^{M}\lambda_{i}=1$, and the weight factor $\lambda_{i}>0$ emphasizes the different priority for harvesting energy at $SR_{i}$. Constraint Equation (6b) corresponds to the power constraint of the ST. Constraint Equation (6c) is corresponds to the SINR constraint such that $\mathrm{SIN}R_{i}$ at $SR_{i}$ is larger than the minimum required SINR, $a_{i}$. Moreover, constraint Equation (6d) shows that the interference power at which the ST interferes with $PU_{l}$ should be lower than $I_{t}$ in the underlay cognitive mode. Since the problem is non-convex due to the objective function and constraint Equation (6c), it is quite challenging to solve the WSHE problem directly. Therefore, in the next sub-section we propose an algorithm to solve the WSHE problem based on semidefinite relaxation and particle swarm optimization.

### 3.1. SDR Approach with Fixed PS Ratios

The idea for solving problem (6) is to separate the variables into two groups including the precoding vectors $\left\{w_{i}\right\}$ and the power splitting ratios $\left\{\theta_{i}\right\}$. To do this, we transform the objective function in Equation (6a) into:

$$\max_{0<\theta_{i}<1,\forall i\;}\left(\max_{\left\{w_{i}\;\right\}}\;\;\sum_{i=1}^{M}\lambda_{i}EH_{i}\right).$$

After that, let us consider problem (7) with fixed PS ratios $\left\{\theta_{i}\right\}$ and only beamforming vector variables as follows:

(7a) $$\begin{array}{cc} \max_{\left\{w_{i}\right\}}\;\;\;\; & \sum_{i=1}^{M}\lambda_{i}EH_{i} \end{array}$$

(7b) $$\begin{array}{cc} s.t.\;\;\; & \mathrm{Equations}\;(6a),\;(6c)\;\mathrm{and}\;(6d). \end{array}$$

Using the semidefinite relaxation method [20], we convert Equation (7) into standard semidefinite programming (SDP) [26] which can be effectively resolved via general-purpose numerical solver like CVX [21]. For this, let us denote $W_{i}=w_{i}w_{i}^{H}$, $H_{i}=h_{i}h_{i}^{H}$, ∀i and $G_{l}=g_{l}g_{l}^{H}$, ∀l. Based on the formulas u=Tr(u), ${∥u∥}^{2}=u^{H}u$, and $\mathrm{Tr}\left(\mathrm{UV}\right)=\mathrm{Tr}\left(\mathrm{VU}\right)$, the following results are obtained as follows:

$${∥w_{i}∥}^{2}=w_{i}^{H}w_{i}=\mathrm{Tr}\left(w_{i}^{H}w_{i}\right)=\mathrm{Tr}\left(W_{i}\right)$$

$${\left|h_{i}^{H}w_{j}\right|}^{2}=\mathrm{Tr}\left(w_{j}^{H}h_{i}h_{i}^{H}w_{j}\right)=\mathrm{Tr}\left(H_{i}W_{j}\right).$$

Similarly, ${\left|g_{l}^{H}w_{i}\right|}^{2}=\mathrm{Tr}\left(G_{l}W_{i}\right)$. We also have the property as $W_{i}\;=\;w_{i}w_{i}^{H}\Leftrightarrow W_{i}⪰0$ and $\mathrm{rank}\left(W_{i}\right)=1$. Therefore, the problem (7) can be recast as follows:

(8a) $$\begin{array}{c} \min_{\left\{W_{i}\right\}}\left(-\sum_{i=1}^{M}\lambda_{i}\eta_{i}\left(1-\theta_{i}\right)\left(\sum_{j=1}^{M}\mathrm{Tr}\left(H_{i}W_{j}\right)+\sigma_{n}^{2}\right)\right) \end{array}$$

(8b) $$\begin{array}{c} \;\;s.t.\;\;\sum_{i=1}^{M}\mathrm{Tr}\left(W_{i}\right)-P_{\max}\leq 0 \end{array}$$

(8c) $$\begin{array}{c} -\frac{\mathrm{Tr}\left(H_{i}W_{i}\right)}{a_{i}}+\sum_{j\neq i}^{M}\mathrm{Tr}\left(H_{i}W_{j}\right)+\sigma_{n}^{2}+\frac{\sigma_{v}^{2}}{\theta_{i}}\leq 0,\;\forall i \end{array}$$

(8d) $$\begin{array}{c} \;\;\;\;\;\;\;\;\;\sum_{i=1}^{M}\mathrm{Tr}\left(G_{l}W_{i}\right)\;-\;I_{t}\leq \;0,\forall l \end{array}$$

(8e) $$\begin{array}{c} \;\;\;\;\;\;\;\;\;W_{i}⪰0,\;\forall i \end{array}$$

(8f) $$\begin{array}{c} \;\;\;\;\;\;\;\;\;\mathrm{rank}\left(W_{i}\right)=1,\;\forall i. \end{array}$$

The above optimization problem is non-convex due to constraint (8f). According to the SDR technique, we remove constraint (8f) to make problem (8) a standard semidefinite programming (SDP) problem. Here, we define problem (8) without constraint (8f) as the problem (8)-SDR. Then, we solve the problem (8)-SDR via a numerical solver for the convex optimization problem. Note that we refer to Remark 2 for convex problem with respect to complex-valued variables. However, the general SDP problem does not always give the rank-1 solutions and there is also no general method to prove the rank-1 solutions. In [27], the uplink-downlink duality is exploited to obtain the rank-1 solution for the SDP problem with *M* separable matrix variables and *M* linear constraints. The idea is to convert the downlink problem into the virtual uplink problem and then construct the rank-1 solution. However, this method can not be applied to our problem due to the different objective function of weighted sum harvested energy and the appearance of the constraints of total transmit power limit and interference threshold at primary users. In [28], in particular, the rank-1 solution of the SDP problem is always obtained whenever M≤L+2 with the number of matrix variables *M* and the number of constraints *L*. However, this result can not be applied to our proposed SDP problem since the number of constraints and the number of variables in problem (8)-SDR do not satisfy the above rank-1 conditions whenever the number of primary users is larger than 1. Interestingly, the optimal solution to problem (8)-SDR can be proven as rank-1. Therefore, it is also the optimal solution of the original problem (8).

(9) LWi,α,βi,γl,Ei=−∑i=1Mλiηi1−θi∑j=1MTrHiWj+σn2+α∑i=1MTrWi−Pmax+∑i=1Mβi−11aiaiTrHiWi+∑j≠iMTrHiWj+σn2+σv2θi+∑l=1Lγl∑i=1MTrGlWi−It−∑i=1MTrEiWi.

**Lemma** **1.** *The optimal solution, $W_{i}$, of problem (8)-SDR is rank-1, i.e., $\mathrm{rank}\left(W_{i}\right)=1,\;\forall i=1,...,M$*.

**Proof.**  The Karush–Kuhn–Tucker (KKT) optimality conditions are applied to the proof of Lemma 1. The Lagrangian function of problem (8)-SDR is written as seen in (9) where α≥0, $\beta_{i}\geq 0,\;\forall i$, $\gamma_{l}\geq 0,\;\forall l$, and $E_{i}⪰0,\;\forall i$ are the dual variables associated with constraints (8b), (8c), (8d), and (8e), respectively. Since $W_{i}⪰0$, $H_{i}=h_{i}h_{i}^{H}$, and $G_{l}=g_{l}g_{l}^{H}$, we derive that $\mathrm{Tr}\left(W_{i}\right)$, $\mathrm{Tr}\left(H_{j}W_{i}\right)$ and $\mathrm{Tr}\left(G_{l}W_{i}\right)$ are real-valued for all i,j,l. Therefore, the objective function (8a), the constraint functions in (8b), (8c), (8d) and then Lagrangian (9) are real-valued functions with complex-valued Hermitian matrix variables. At the optimal points, the gradient of Lagrangian (9) must vanish at all independent real variables, i.e., all real and imaginary variables of complex-valued matrix variables $\left\{W_{i}\right\}$. This is equivalent to the gradient of real-valued Lagrangian vanishing at $\left\{W_{i}\right\}$ according to Theorem 2 in ([29], Section 4). ☐

Following Lemma 1 in ([30], Section 4B), we obtain the gradient of $\mathrm{Tr}\left(\mathrm{AX}\right)$ as $\nabla_{X}\mathrm{Tr}\left(\mathrm{AX}\right)=\frac{\partial \mathrm{Tr}\left(\mathrm{AX}\right)}{\partial X^{*}}=A$ where A and X are Hermitian matrices. Since $W_{i}$, $H_{j}$, and $G_{l}$ are Hermitian matrices for all i,j,l, we derive the gradient of Lagrangian in (10). As a result, we obtain the KKT optimality conditions used for the proof as follows:

(10) $$\begin{array}{c} -\sum_{j=1}^{M}\lambda_{j}\eta_{j}\left(1-\theta_{j}\right)H_{j}+\alpha I-\frac{\beta_{i}}{a_{i}}H_{i}+\sum_{j\neq i}^{M}\beta_{j}H_{j}\\ +\sum_{l=1}^{L}\gamma_{l}G_{l}-E_{i}=0,\;\forall i\;\;\;\;\;\;\;\;\;\;\;\;\;\;\;\;\;\;\;\;\;\;\;\;\;\;\;\; \end{array}$$

(11) $$E_{i}W_{i}\;=\;0,\;\forall i$$

(12) $$\alpha ,\;\beta_{i},\;\gamma_{l}\geq 0;\;E_{i},\;W_{i}⪰0,\;\forall i,l,$$

where (10) and (11), respectively, are calculated from $\nabla_{W_{i}}L=0$ and $\mathrm{Tr}\left(E_{i}W_{i}\right)=0$ with $E_{i},\;W_{i}⪰0$. In addition, $I\in C^{N\times N}$ is an identity matrix. From (10), we derive

(13) $$E_{i}=\;A-\left(\beta_{i}+\frac{\beta_{i}}{a_{i}}\right)H_{i},\;\forall i,$$

where

(14) $$A=\alpha I+\sum_{j=1}^{M}\beta_{j}H_{j}+\sum_{l=1}^{L}\gamma_{l}G_{l}-\;\sum_{j=1}^{M}\lambda_{j}\eta_{j}\left(1-\theta_{j}\right)H_{j}.$$

First, α>0 is proved by contradiction. Assuming that α=0, let us introduce

$$D_{i}=\left[h_{1},...,h_{i-1},h_{i+1},...,h_{M},g_{1},...,g_{L}\right],\;\forall i.$$

Since $D_{i}\in C^{N\times (M+L-1)}$, we set a basic of $\mathrm{Null}\left(D_{i}^{H}\right)$ as $L_{i}\in C^{N\times (N-M-L+1)}$. In this paper, the number of antennas *N* is assumed to be larger than M+L−1. Thus, we derive $H_{j}L_{i}=0,\;\forall j\neq i,$ and $G_{l}L_{i}=0,\;\forall i,l$. After that, with $x\in C^{(N-M-L+1)\times 1}$ and $y=L_{i}x$, we obtain

(15) $$y^{H}E_{i}y=-\left(\lambda_{i}\eta_{i}\left(1-\theta_{i}\right)+\frac{\beta_{i}}{a_{i}}\right)y^{H}H_{i}y$$

(16) $$y^{H}E_{i}y=-\left(\lambda_{i}\eta_{i}\left(1-\theta_{i}\right)+\frac{\beta_{i}}{a_{i}}\right){\left|h_{i}^{H}L_{i}x\right|}^{2}\leq 0.$$

Owing to $E_{i}⪰0$ and (16), we obtain the following:

(17) $$y^{H}E_{i}y\geq 0,\;\forall y\;\Rightarrow h_{i}^{H}L_{i}x=0,\;\forall x\;\Rightarrow \;h_{i}^{H}L_{i}=0.$$

From the fact that the transmission channels $h_{i},g_{l},\;\forall i,l$, are independent and random, we can assume that $h_{i}\notin \mathrm{Range}\left(D_{i}\right)$. Thus, we derive $h_{i}^{H}L_{i}\neq 0$, which contradicts (17). Subsequently, α>0 is satisfied. Moreover, since $G_{l}⪰0$, we have

(18) $$\left(\alpha I+\sum_{l=1}^{L}\gamma_{l}G_{l}\right)≻0.$$

Second, we also prove that A≻0 by contradiction. We assume that A⊁0, i.e., there exists at least a vector z≠0 such that $z^{H}\mathrm{Az}\leq 0$. From (13), we derive

(19) $$z^{H}E_{i}z=\;z^{H}\mathrm{Az}-\left(\beta_{i}+\frac{\beta_{i}}{a_{i}}\right)z^{H}H_{i}z,\;\forall i.$$

With $E_{i},H_{i}⪰0$, we have $z^{H}E_{i}z\geq 0$, and $z^{H}H_{i}z\geq 0$. Thus, from (19) we derive $z^{H}Az=0$, and $z^{H}H_{i}z=0,\;\forall i$. From (14), we derive $z^{H}\left(\alpha I+\sum_{l=1}^{L}\gamma_{l}G_{l}\right)z=0$, which contradicts (18). It thus follows that A≻0.

Last, we prove that $\mathrm{rank}\left(W_{i}\right)=1,\;\forall i$. We know that rank(X−Y)≥rank(X)−rank(Y) is a basic rank inequality property. Thus, according to (13), we derive the following:

(20) $$\mathrm{rank}\left(E_{i}\right)\geq \mathrm{rank}\left(A\right)-\mathrm{rank}\left(\left(\beta_{i}+\frac{\beta_{i}}{a_{i}}\right)H_{i}\right)=N-1$$

since $\mathrm{rank}\left(A\right)=N$ and $\mathrm{rank}\left(H_{i}\right)=1$. From (11), we have $\mathrm{Range}\left(W_{i}\right)\subseteq \mathrm{Null}\left(E_{i}\right)$, so $\mathrm{rank}\left(W_{i}\right)\leq N-\mathrm{rank}\left(E_{i}\right)$. Then, from (20) we derive $\mathrm{rank}\left(W_{i}\right)\leq 1$. Since $W_{i}=0$ does not satisfy constraint (8c), we can finally conclude that $\mathrm{rank}\left(W_{i}\right)=1$. Thus, Lemma 1 is completely proven.

**Remark** **1.** *Inspired by the idea in [3], we can propose another approach to prove Lemma 1 by considering the minimization of the partial Lagrangian and the dual problem. First, we have the partial Lagrangian of the primal problem (8)-SDR expressed as follows:*

(21) LWi,α,βi,γl=−∑i=1Mλiηi1−θi∑j=1MTrHiWj+σn2+α∑i=1MTrWi−Pmax+∑i=1Mβi−11aiaiTrHiWi+∑j≠iMTrHiWj+σn2+σv2θi+∑l=1Lγl∑i=1MTrGlWi−It

*where α≥0, $\beta_{i}\geq 0,\;\forall i$, and $\gamma_{l}\geq 0,\;\forall l$ are the dual variables associated with constraints (8b), (8c), and (8d), respectively. The dual function of dual problem is expressed as*

(22) $$g\left(\alpha ,\left\{\beta_{i}\right\},\left\{\gamma_{l}\right\}\right)=\min_{W_{i}⪰0,\forall i}\;L\left(\left\{W_{i}\right\},\alpha ,\left\{\beta_{i}\right\},\left\{\gamma_{l}\right\}\right).$$

*The dual problem is expressed as follows:*

(23a) $$\begin{array}{cc} \max_{\alpha ,\left\{\beta_{i}\right\},\left\{\gamma_{l}\right\}} & g\left(\alpha ,\left\{\beta_{i}\right\},\left\{\gamma_{l}\right\}\right) \end{array}$$

(23b) $$\begin{array}{cc} s.t.\;\;\;\; & \alpha \geq 0,\;\beta_{i}\geq 0,\;\gamma_{l}\geq 0,\;\forall i,l. \end{array}$$

*We rewrite the partial Lagrangian function and equivalently obtain the minimization problem as:*

(24) $$\min_{W_{i}⪰0,\forall i}\sum_{i=1}^{M}\mathrm{Tr}\left(\left(-\;\sum_{j=1}^{M}\lambda_{j}\eta_{j}\left(1-\theta_{j}\right)H_{j}+\alpha I-\frac{\beta_{i}}{a_{i}}H_{i}+\sum_{j\neq i}^{M}\beta_{j}H_{j}+\sum_{l=1}^{L}\gamma_{l}G_{l}\right)W_{i}\right).$$

*That is equivalent to the optimization problem for separate variables $W_{i}$:*

(25) $$\min_{W_{i}⪰0}\mathrm{Tr}\left(F_{i}W_{i}\right),\;\forall i$$

*where we denote $F_{i}=-\;\sum_{j=1}^{M}\lambda_{j}\eta_{j}\left(1-\theta_{j}\right)H_{j}+\alpha I-\frac{\beta_{i}}{a_{i}}H_{i}+\sum_{j\neq i}^{M}\beta_{j}H_{j}+\sum_{l=1}^{L}\gamma_{l}G_{l},\;\forall i$. For simple notation, we denote $\alpha ,\left\{\beta_{i}\right\},\left\{\gamma_{l}\right\}$ as the optimal dual solution of (23). Then, we derive $F_{i}⪰0$ by contradiction. Suppose that $F_{i}$ is not a positive semidefinite matrix, i.e., there exists at least a vector x≠0 such that $x^{H}F_{i}x<0$. Therefore, if we choose $W_{i}=txx^{H}$ where t>0, then $\mathrm{Tr}\left(F_{i}W_{i}\right)=\mathrm{Tr}\left(F_{i}txx^{H}\right)=tx^{H}F_{i}x<0$ is unbounded below when t→+∞. Therefore, we can not obtain the bounded optimal dual value. Hence, $F_{i}⪰0$. From (25), we derive $\mathrm{Tr}\left(F_{i}W_{i}\right)=0$ and then $F_{i}W_{i}=0$ since $F_{i}⪰0$ and $W_{i}⪰0$, ∀i. The rest of the rank-1 proof is similar to the KKT method from Equation (13) where $F_{i}$ has the same role as $E_{i}$ in (13).*

**Remark** **2.** *The real-valued objective and constraint functions with complex-valued variables in the problem (8)-SDR exist with partial derivatives according to Definition 2.2 ([31], Chapter 2) or Theorem 1 ([32], Section 2). These functions are affine and convex in complex variables $W_{i}$ according to Remark 3.15 ([33], Chapter 3). Thus, the problem (8)-SDR is a convex optimization problem with respect to complex-valued matrix variables. Moreover, KKT conditions can be applied to the problem (8)-SDR with real-valued functions and complex-valued variables from Remark 9.13 ([33], Chapter 9). Another approach to solving problem (8)-SDR is to convert problems with complex-valued variables to equivalent problem with real-valued variables according to Remark 1.20 ([33], Chapter 1). However, the proof of rank-1 constraints which we have not obtained yet, is still much more complicated than that of the problem (8)-SDR with complex-valued variables, although the same optimal values are achieved and the rank-1 constraints are satisfied in numerical experiments*.

### 3.2. PSO-SDR Approach to Maximizing WSHE

We can apply the brute-force method to search the optimal PS ratios over all possible collections of $\left\{\theta_{i}\right\}$. Since the brute-force search method has very high computational complexity, i.e., in the *M*-dimension search, it is very slow to obtain the optimal results. To avoid this difficulty, a PSO-based algorithm [22,34,35] with low complexity, high convergence rate, and high rigor is exploited to search the best power-splitting ratios in the paper.

A main description of the PSO-based algorithm is presented as follows. The limited number of iterations and the number of elements in a swarm are denoted by $T_{\max}$ and $N_{S}$, respectively. We assign each element’s position to a set of *M* PS ratios $\left(\theta_{1},...,\theta_{M}\right)$. By observing the SINR constraint (8c) in problem (8), we do not obtain this constraint if $\theta_{i}$ is extremely small. As a result, we set a lowest value for $\theta_{i}$, represented by $\theta_{\min}$. Therefore, $\left[\theta_{\min},1\right]$ is the search interval of each PS ratio $\theta_{i}$. For the *n*-th particle, $x_{n}$, $v_{n}$, and $p_{b,n}$, are represented for its position, velocity and local optimal position, respectively. The global best position denoted as $g_{b}$ is obtained by collecting information from all the particles. With the set of PS ratios, $x_{n}$, we solve problem (8) with the SDR technique to achieve the maximum WSHE value denoted as $f\left(x_{n}\right)$. For the update step, the new velocity is affected by the previous velocity with the inertia weight, $i_{w}$, and the local and global best positions with the cognitive and social factors, denoted as $c_{1}$ and $c_{2}$, respectively. Finally, problem (6) is solved by the proposed PSO-based algorithm described in detail in Table 2.

We can calculate the computational complexity of the PSO-based algorithm according to $T_{\max}$, $N_{S}$, and the complexity in solving the SDP problem (8)-SDR. In the PSO method, the problem (8)-SDR is solved within $T_{\max}N_{S}$ times. In the problem (8)-SDR, there exist $\left(M+L+1\right)$ linear constraints for *M* matrix variables, and each matrix variable has a size N×N. Therefore, the complexity of solving problem (8)-SDR is OMNM3N6+M+L+1MN2log11ζζ [36]. As a result, the computational complexity of the proposed algorithm is OTmaxNSMNM3N6+M+L+1MN2log11ζζ, for a given solution accuracy ζ.

The proposed solutions are not only suitable for the conventional cognitive radio (CR) network but also CR sensor networks where the secondary receivers are the wireless sensor nodes with a limited energy and low complexity due to the following reason. After the channel side information (CSI) from the secondary transmitter to the secondary receivers and primary users is obtained, the proposed SDR-PSO algorithm in Table 2 is performed at the secondary transmitter, which is usually a base station equipped with powerful computational processors. After that, the optimal power-splitting ratios are sent to the secondary receivers for setting the PS ratios by the secondary transmitter. As a result, the secondary receivers can split the incoming signal by optimal PS ratios without running the algorithms. Therefore, the proposed algorithms can be applied to low resources-constrained CR sensor networks.

### 3.3. Zero-Forcing Beamforming (ZFBF) Problem 

In the ZFBF problem, we choose the weighted vector for the information message to the $SR_{i}$ which are orthogonal to the channels from the ST to the other $SR_{j}$, ∀j≠i, and the PUs, i.e., $w_{i}\;\;\in \;\;Null\left(B_{i}^{H}\right)$ where $B_{i}=\left[h_{1},...,h_{i-1},h_{i+1},...,h_{M},g_{1},...,g_{L}\right]\in C^{N\times (M+L-1)}$. The null-space of $B_{i}^{H}$ has an orthonormal basic $Q_{i}\in C^{N\times \left(N-(M+L-1)\right)}$ where $Q_{i}^{H}Q_{i}=I$. As a result, $w_{i}$ can be expressed $w_{i}=Q_{i}u_{i}$ where $u_{i}\in C^{(N-(M+L-1))\times 1}$. Let us introduce $U_{i}=u_{i}u_{i}^{H}$. Some calculations are then performed as follows:

(26a) $$\begin{array}{c} \mathrm{Tr}\left(W_{i}\right)=\mathrm{Tr}\left(Q_{i}u_{i}u_{i}^{H}Q_{i}^{H}\right)=\mathrm{Tr}\left(Q_{i}^{H}Q_{i}u_{i}u_{i}^{H}\right)=\mathrm{Tr}\left(U_{i}\right),\forall i \end{array}$$

(26b) $$\begin{array}{c} \mathrm{Tr}\left(H_{i}W_{i}\right)=\mathrm{Tr}\left(H_{i}Q_{i}u_{i}u_{i}^{H}Q_{i}^{H}\right)=\mathrm{Tr}\left(Q_{i}^{H}H_{i}Q_{i}U_{i}\right),\forall i \end{array}$$

(26c) $$\begin{array}{c} \mathrm{Tr}\left(H_{j}W_{i}\right)=0,\mathrm{Tr}\left(G_{l}W_{i}\right)=0,\forall j\neq i,\forall l. \end{array}$$

As a result, the optimization problem with ZFBF beamforming is formulated as follows:

(27a) $$\begin{array}{cc} \max_{\left\{U_{i}\right\},\left\{\theta_{i}\right\}} & \sum_{i=1}^{M}\lambda_{i}\eta_{i}\left(1-\theta_{i}\right)\left(\mathrm{Tr}\left(Q_{i}^{H}H_{i}Q_{i}U_{i}\right)+\sigma_{n}^{2}\right) \end{array}$$

(27b) $$\begin{array}{cc} s.t.\;\;\; & \sum_{i=1}^{M}\mathrm{Tr}\left(U_{i}\right)-P_{\max}\leq 0 \end{array}$$

(27c) $$\begin{array}{c} -\frac{\mathrm{Tr}\left(Q_{i}^{H}H_{i}Q_{i}U_{i}\right)}{a_{i}}+\sigma_{n}^{2}+\frac{\sigma_{v}^{2}}{\theta_{i}}\leq 0,\forall i \end{array}$$

(27d) $$\begin{array}{c} U_{i}⪰0,0<\theta_{i}<1,\forall i \end{array}$$

(27e) $$\begin{array}{c} \mathrm{rank}\left(U_{i}\right)=1. \end{array}$$

The ZFBF problem is solved by using the similar solution of the general problem (6). The optimal value of this ZFBF problem is compared with the optimal WSHE value of (6) in the numerical section.

### 3.4. Equal Power Splitting (EPS) Problem

In the special case of EPS design, the power-splitting factors $\theta_{i}$ are simply assigned to be 0.5, ∀i. It means that the secondary receivers share a half received signal energy for decoding information and a half for harvesting power. The secondary transmitter can control the direction and power of beamforming vectors to achieve the WSHE maximization without join to the secondary receivers. Thus, the EPS problem is formulated as follows:

(28a) $$\begin{array}{cc} \min_{\left\{W_{i}\right\}\;}\;\;\;\; & \left(-\;0.5\sum_{i=1}^{M}\lambda_{i}\eta_{i}\left(\sum_{j=1}^{M}\mathrm{Tr}\left(H_{i}W_{j}\right)\;+\;\sigma_{n}^{2}\right)\right) \end{array}$$

(28b) $$\begin{array}{cc} s.t.\;\;\; & \sum_{i=1}^{M}\mathrm{Tr}\left(U_{i}\right)-P_{\max}\leq 0 \end{array}$$

(28c) $$\begin{array}{c} -\frac{\mathrm{Tr}\left(H_{i}W_{i}\right)}{a_{i}}\;\;+\;\sum_{j\neq i}^{M}\mathrm{Tr}\left(H_{i}W_{j}\right)+\sigma_{n}^{2}\;\;+\frac{\sigma_{v}^{2}}{0.5}\;\leq \;\;0,\forall i \end{array}$$

(28d) $$\begin{array}{c} \sum_{i=1}^{M}\mathrm{Tr}\left(G_{l}W_{i}\right)\;-\;I_{t}\leq \;0,\forall l \end{array}$$

(28e) $$\begin{array}{c} W_{i}⪰0,\forall i \end{array}$$

(28f) $$\begin{array}{c} \mathrm{rank}\left(W_{i}\right)=1,\forall i. \end{array}$$

For the EPS problem with fixed PS factors, we only have the variables of beamforming vectors in the optimization problem. Therefore, we can obtain the optimal solution by SDR technique and CVX solver. The optimal WSHE value in the EPS problem is considered as a special case in comparision with the general WSHE problem (6).

## 4. Robust WSHE Maximization with Imperfect Channels

The perfect CSI assumption is not practical due to many reasons such as the mobility of users, the estimation errors, limited CSI feedback quantization, etc. Therefore, we propose a robust design for the imperfect CSI of the channels. First, the models of imperfect CSI from the ST to the SRs and the PUs, are respectively considered as follows:

$$h_{i}={\hat{h}}_{i}+\Delta h_{i},\Delta h_{i}\in C^{N\times 1},∥\Delta h_{i}∥\leq \varepsilon_{i}$$

$$g_{l}={\hat{g}}_{l}+\Delta g_{l},\Delta g_{l}\in C^{N\times 1},∥\Delta g_{l}∥\leq \delta_{l},$$

where $h_{i}$ and $g_{l}$ are the actual channels; ${\hat{h}}_{i}$ and ${\hat{g}}_{l}$ are the estimated channels at the ST; and $\Delta h_{i}$ and $\Delta g_{l}$ are the CSI errors. The error bounds, $\varepsilon_{i}$ and $\delta_{l}$, represent the radius of the uncertainty region of the estimated CSI channels. When these radiuses go to zero, the estimated channels become the perfect channels. For the imperfect CSI case, the optimization problem (6) becomes non-convex and has infinite number of constraints. The idea to solve this problem is to apply the vector inequalities and the S-Procedure [26,33] in order to obtain a tractable SDP problem. The robust WSHE maximization problem can be expressed as problem (29).

(29a) $$\begin{array}{cc} \max_{\left\{w_{i}\right\},\left\{\theta_{i}\right\}}\;\;\;\;\; & \min_{∥\Delta h_{i}∥\leq \varepsilon_{i},\forall i}\sum_{i=1}^{M}\lambda_{i}\eta_{i}\left(1-\theta_{i}\right)\left(\sum_{j=1}^{M}{\left|\left({\hat{h}}_{i}^{H}+\Delta h_{i}^{H}\right)w_{j}\right|}^{2}+\sigma_{n}^{2}\right) \end{array}$$

(29b) $$\begin{array}{cc} s.t.\;\;\;\;\;\;\;\;\; & \sum_{i=1}^{M}\mathrm{Tr}\left(W_{i}\right)\leq P_{\max} \end{array}$$

(29c) $$\begin{array}{c} \frac{\theta_{i}{\left|\left({\hat{h}}_{i}^{H}+\Delta h_{i}^{H}\right)w_{i}\right|}^{2}}{\theta_{i}\left(\sum_{j=1,j\neq i}^{M}{\left|\left({\hat{h}}_{i}^{H}+\Delta h_{i}^{H}\right)w_{j}\right|}^{2}\;+\;\sigma_{n}^{2}\right)+\sigma_{v}^{2}}\;\geq \;a_{i}\;,\;\;\forall ∥\Delta h_{i}∥\leq \varepsilon_{i},\;\forall i \end{array}$$

(29d) $$\begin{array}{c} \sum_{i=1}^{M}{\left|\left({\hat{g}}_{l}^{H}+\Delta g_{l}^{H}\right)w_{i}\right|}^{2}\leq \;\;I_{t},\;\forall ∥\Delta g_{l}∥\leq \delta_{l},\;\;\forall l \end{array}$$

(29e) $$\begin{array}{c} 0<\theta_{i}<1,\;\forall i. \end{array}$$

First, we consider the objective function (29a) as follows

$$\min_{∥\Delta h_{i}∥\leq \varepsilon_{i},\forall i}\sum_{i=1}^{M}\lambda_{i}\eta_{i}\left(1-\theta_{i}\right)\left(\sum_{j=1}^{M}{\left|\left({\hat{h}}_{i}^{H}+\Delta h_{i}^{H}\right)w_{j}\right|}^{2}+\sigma_{n}^{2}\right)$$

From the inequalities $\left|x+y\right|\geq \left|x\right|-\left|y\right|$ and $\left|u^{H}v\right|\leq ∥u∥∥v∥$, we obtain the results as follows

$$\begin{array}{c} \left|\left({\hat{h}}_{i}^{H}+\Delta h_{i}^{H}\right)w_{j}\right|\geq \left|{\hat{h}}_{i}^{H}w_{j}\right|-\left|\Delta h_{i}^{H}w_{j}\right|\\ \;\;\;\;\;\;\;\;\;\;\;\;\;\;\;\;\;\;\;\;\;\;\;\;\;\;\;\;\;\;\;\;\;\;\;\;\;\;\;\;\;\;\;\;\;\;\;\;\;\;\;\geq \left|{\hat{h}}_{i}^{H}w_{j}\right|-\varepsilon_{i}∥w_{j}∥. \end{array}$$

Subsequently, we have

$${\left|\left({\hat{h}}_{i}^{H}+\Delta h_{i}^{H}\right)w_{j}\right|}^{2}\geq {\left(\left|{\hat{h}}_{i}^{H}w_{j}\right|-\varepsilon_{i}∥w_{j}∥\right)}^{2}$$

$$\begin{array}{c} {\left(\left|{\hat{h}}_{i}^{H}w_{j}\right|-\varepsilon_{i}∥w_{j}∥\right)}^{2}={\left|{\hat{h}}_{i}^{H}w_{j}\right|}^{2}+\varepsilon_{i}^{2}{∥w_{j}∥}^{2}\;\;\;\;\;\;\;\;\;\\ \;\;\;\;\;\;\;\;\;\;\;\;\;\;\;\;\;\;\;\;\;\;\;\;\;\;\;\;\;\;\;\;\;\;\;\;\;\;\;\;\;\;\;\;\;\;\;\;\;\;\;\;\;\;\;\;\;\;\;\;\;\;\;\;\;\;-2\varepsilon_{i}\left|{\hat{h}}_{i}^{H}w_{j}\right|∥w_{j}∥ \end{array}$$

$$\begin{array}{c} {\left(\left|{\hat{h}}_{i}^{H}w_{j}\right|-\varepsilon_{i}∥w_{j}∥\right)}^{2}\geq w_{j}^{H}{\hat{h}}_{i}{\hat{h}}_{i}^{H}w_{j}+\varepsilon_{i}^{2}w_{j}^{H}w_{j}\\ \;\;\;\;\;\;\;\;\;\;\;\;\;\;\;\;\;\;\;\;\;\;\;\;\;\;\;\;\;\;\;\;\;\;\;\;\;\;\;\;\;\;\;\;\;\;\;\;\;\;\;\;\;\;\;\;\;\;\;\;\;\;\;\;\;-2\varepsilon_{i}∥{\hat{h}}_{i}∥{∥w_{j}∥}^{2} \end{array}$$

$${\left|\left({\hat{h}}_{i}^{H}+\Delta h_{i}^{H}\right)w_{j}\right|}^{2}\geq w_{j}^{H}{\tilde{H}}_{i}w_{j},$$

where ${\tilde{H}}_{i}={\hat{h}}_{i}{\hat{h}}_{i}^{H}+\left(\varepsilon_{i}^{2}-2\varepsilon_{i}∥{\hat{h}}_{i}∥\right)I$. Thus, the objective function is recast as follows

(30) $$\max_{\left\{w_{i}\right\},\left\{\theta_{i}\right\}}\;\;\sum_{i=1}^{M}\lambda_{i}\eta_{i}\left(1-\theta_{i}\right)\left(\sum_{j=1}^{M}w_{j}^{H}{\tilde{H}}_{i}w_{j}+\sigma_{n}^{2}\right).$$

Next, we apply S-Procedure to convert the SINR and PU constraints to linear matrix inequality (LMI) constraints.

**Lemma** **2.** *(S-Procedure) We have the function*

fkx=xHAkx+2RebkHx+ck,

*where k=1,2, $A_{k}\in H^{N}$, $b_{k}\in C^{N\times 1}$, and $c_{k}\in C$. Then, the implication $f_{1}\left(x\right)\leq 0\Rightarrow f_{2}\left(x\right)\leq 0$ holds if and only if there exists a ξ≥0 such that*

(31) $$\xi \left[\begin{array}{cc} A_{1} & b_{1}\\ b_{1}^{H} & c_{1} \end{array}\right]-\left[\begin{array}{cc} A_{2} & b_{2}\\ b_{2}^{H} & c_{2} \end{array}\right]⪰0,$$

*provided that there exists a point $\bar{x}$ such that $f_{1}\left(\bar{x}\right)<0$.*

We first analyze the SINR constraints (29c) at the SRs as follows:

f1Δhi=ΔhiHIΔhi+2Re0HΔhi+−εi2≤0

$$\begin{array}{c} f_{2}\left(\Delta h_{i}\right)=-\frac{\left({\hat{h}}_{i}^{H}+\Delta h_{i}^{H}\right)w_{i}w_{i}^{H}\left({\hat{h}}_{i}+\Delta h_{i}\right)}{a_{i}}+\left({\hat{h}}_{i}^{H}+\Delta h_{i}^{H}\right)\\ \;\;\;\;\;\;\;\;\;\;\;\;\;\;\;\;\;\;\;\;\;\;\;\;\;\;\;\;\left(\sum_{j\neq i}^{M}w_{j}w_{j}^{H}\right)\left({\hat{h}}_{i}+\Delta h_{i}\right)+\sigma_{n}^{2}+\frac{\sigma_{v}^{2}}{\theta_{i}}\leq 0. \end{array}$$

We can rewrite $f_{2}\left(\Delta h_{i}\right)$ as follows

f2Δhi=ΔhiHAiΔhi+2ReAih^iHΔhi+h^iHAih^i+σn2+σv2θi≤0,

where $A_{i}=\sum_{j\neq i}^{M}w_{j}w_{j}^{H}-\frac{w_{i}w_{i}^{H}}{a_{i}}$. Note that $A_{i}$, ∀i, are Hermitian matrices.

According to Lemma 2, there exists $\alpha_{i}\geq 0$ satisfying

$$\alpha_{i}\left[\begin{array}{cc} I & 0\\ 0 & -\varepsilon_{i}^{2} \end{array}\right]-\left[\begin{array}{cc} A_{i} & A_{i}{\hat{h}}_{i}\\ {\hat{h}}_{i}^{H}A_{i} & {\hat{h}}_{i}^{H}A_{i}{\hat{h}}_{i}+\sigma_{n}^{2}+\frac{\sigma_{v}^{2}}{\theta_{i}} \end{array}\right]⪰0$$

(32) $$\Leftrightarrow \left[\begin{array}{cc} \alpha_{i}I-A_{i} & -A_{i}{\hat{h}}_{i}\\ -{\hat{h}}_{i}^{H}A_{i} & -\alpha_{i}\varepsilon_{i}^{2}-\left({\hat{h}}_{i}^{H}A_{i}{\hat{h}}_{i}+\sigma_{n}^{2}+\frac{\sigma_{v}^{2}}{\theta_{i}}\right) \end{array}\right]⪰0,\forall i.$$

Finally, we consider the interference constraints (29d) at the PUs as follows:

f1Δgl=ΔglHIΔgl+2Re0HΔgl+−δl2≤0

f2Δgl=ΔglHBΔgl+2ReBg^lHΔgl+g^lHBg^l−It≤0,

where $B=\sum_{i=1}^{M}w_{i}w_{i}^{H}$. Note that B is Hermitian matrix.

According to Lemma 2, there exists $\beta_{l}\geq 0$ satisfying

$$\beta_{l}\left[\begin{array}{cc} I & 0\\ 0 & -\delta_{l}^{2} \end{array}\right]-\left[\begin{array}{cc} B & B{\hat{g}}_{l}\\ {\hat{g}}_{l}^{H}B & {\hat{g}}_{l}^{H}B{\hat{g}}_{l}-I_{t} \end{array}\right]⪰0,\forall l$$

(33) $$\Leftrightarrow \left[\begin{array}{cc} \beta_{l}I-B & -B{\hat{g}}_{l}\\ -{\hat{g}}_{l}^{H}B & -\beta_{l}\delta_{l}^{2}-{\hat{g}}_{l}^{H}B{\hat{g}}_{l}+I_{t} \end{array}\right]⪰0,\forall l.$$

From (30), (32), and (33), we obtain the robust WSHE problem with imperfect CSI channels as follows

(34a) $$\begin{array}{cc} \max_{\left\{w_{i}\right\},\left\{\theta_{i}\right\},\left\{\alpha_{i}\right\},\left\{\beta_{l}\right\}}\;\; & \sum_{i=1}^{M}\lambda_{i}\eta_{i}\left(1-\theta_{i}\right)\left(\sum_{j=1}^{M}w_{j}^{H}{\tilde{H}}_{i}w_{j}+\sigma_{n}^{2}\right) \end{array}$$

(34b) $$\begin{array}{cc} s.t.\;\;\;\;\;\;\;\;\;\;\;\;\;\; & \left(29b),\;(32),\;(33),\;(29e\right) \end{array}$$

(34c) $$\begin{array}{c} \alpha_{i}\geq 0,\beta_{l}\geq 0,\forall i,l. \end{array}$$

We denote $W_{i}=w_{i}w_{i}^{H}$, and transform the robust problem to the equivalent problem as follows

(35a) $$\begin{array}{cc} \max_{\left\{W_{i}\right\},\left\{\theta_{i}\right\},\left\{\alpha_{i}\right\},\left\{\beta_{l}\right\}} & \sum_{i=1}^{M}\lambda_{i}\eta_{i}\left(1-\theta_{i}\right)\left(\sum_{j=1}^{M}\mathrm{Tr}\left({\tilde{H}}_{i}W_{j}\right)+\sigma_{n}^{2}\right) \end{array}$$

(35b) $$\begin{array}{cc} s.t.\;\;\;\;\;\;\;\;\;\;\;\;\;\; & \sum_{i=1}^{M}\mathrm{Tr}\left(W_{i}\right)-P_{\max}\leq 0 \end{array}$$

(35c) $$\begin{array}{c} \left(32\right),A_{i}=\sum_{j\neq i}^{M}W_{j}-\frac{W_{i}}{a_{i}},\forall i \end{array}$$

(35d) $$\begin{array}{c} \left(33\right),B=\sum_{i=1}^{M}W_{i},\forall l \end{array}$$

(35e) $$\begin{array}{c} 0<\theta_{i}<1,\alpha_{i}\geq 0,\beta_{l}\geq 0,\forall i,l \end{array}$$

(35f) $$\begin{array}{c} \mathrm{rank}\left(W_{i}\right)=1,\forall i. \end{array}$$

This optimization problem is solved by an approach similar to the WHSE problem (6) with perfect channels. For fixed power splitting ratios $\theta_{i}$, by relaxing the rank-1 constraints, the robust problem becomes the SDP form and can be solved by numerical solvers. By using the PSO method, we can obtain the optimal value of the robust problem by relaxing the rank-1 constraints. However, since the solutions do not always guarantee the properties of rank-1, we only obtain the upper bound of the robust WSHE value. The rank-1 suboptimal solutions have not found yet in the paper, and are left for future work where the randomization method [20] can be applied.

## 5. Simulation Results

In this section, the Monte Carlo simulations are performed to evaluate the numerical results of the proposed algorithms. The simulation parameters are given by: the number of SRs and PUs, M=2, L=2, respectively; the maximum transmit power of ST, $P_{\max}=30\;\mathrm{dBW}$; the variance of noises, $\sigma_{n}^{2}=-75\;\mathrm{dBm}$, $\sigma_{v}^{2}=-55\;\mathrm{dBm}$; and the energy harvesting efficiency of all SRs, $\eta_{i}=1$, ∀i. The weighted factors for two SRs are given as $\left(\lambda_{1},\lambda_{2}\right)=\left(0.2,0.8\right)$. We assume that the power attenuation of the channels from the ST to the SRs and the PUs are identical and equal, at 50dB. Each entry of channel vectors is randomly generated from i.i.d. Rayleigh flat-fading according to the above power attenuation.

We define the normalized maximum channel estimation error of secondary receivers and primary users as $\varepsilon_{i}^{\mathrm{nor}}=\frac{\varepsilon_{i}}{∥h_{i}∥}$, $\delta_{l}^{\mathrm{nor}}=\frac{\delta_{l}}{∥g_{l}∥}$, ∀i,l. We assume that the normalized maximum channel estimation errors $\varepsilon_{i}^{\mathrm{nor}}=\delta_{l}^{\mathrm{nor}}=0.001$, ∀i,l. For the PSO method in Table 2, according to the experiments in [34,35], the simulation parameters are set as follows: $i_{w}=0.7$, $c_{1}=1.494$, and $c_{2}=1.494$, with which the good convergence can be obtained in the proposed algorithm. Furthermore, the swarm size, the maximum number of iterations, and the minimum value of $\theta_{i}$, are set as $N_{S}=10$, $T_{\max}=30$, and $\theta_{\min}=0.001$, respectively.

**Figure 2:**
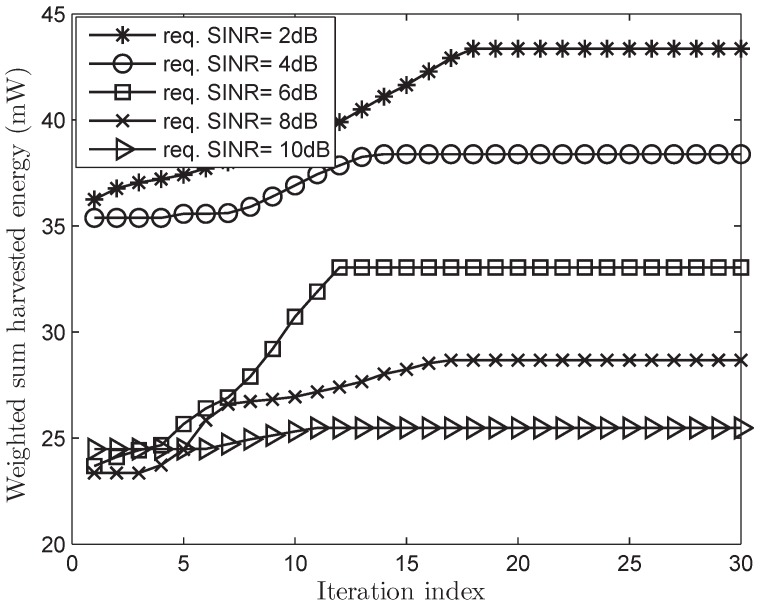
The iteration behavior of the proposed PSO-based algorithm when the required signal-to-interference-plus-noise ratios (SINRs) are given as 2, 4, 6, 8, and 10 dB, respectively and a random perfect CSI is considered.

**Figure 3:**
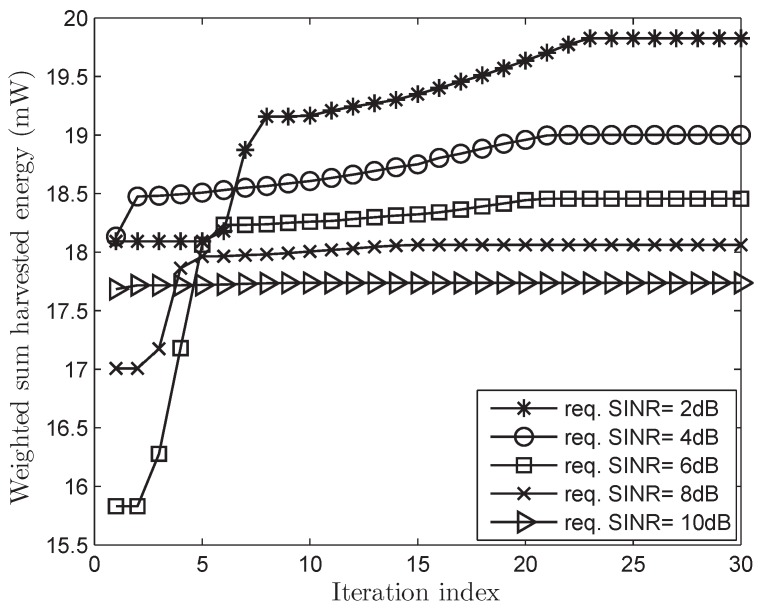
The iteration behavior of the proposed PSO-based algorithm when the required signal-to-interference-plus-noise ratios (SINRs) are given as 2, 4, 6, 8, and 10 dB, respectively, and a random imperfect CSI is considered.

**Figure 4:**
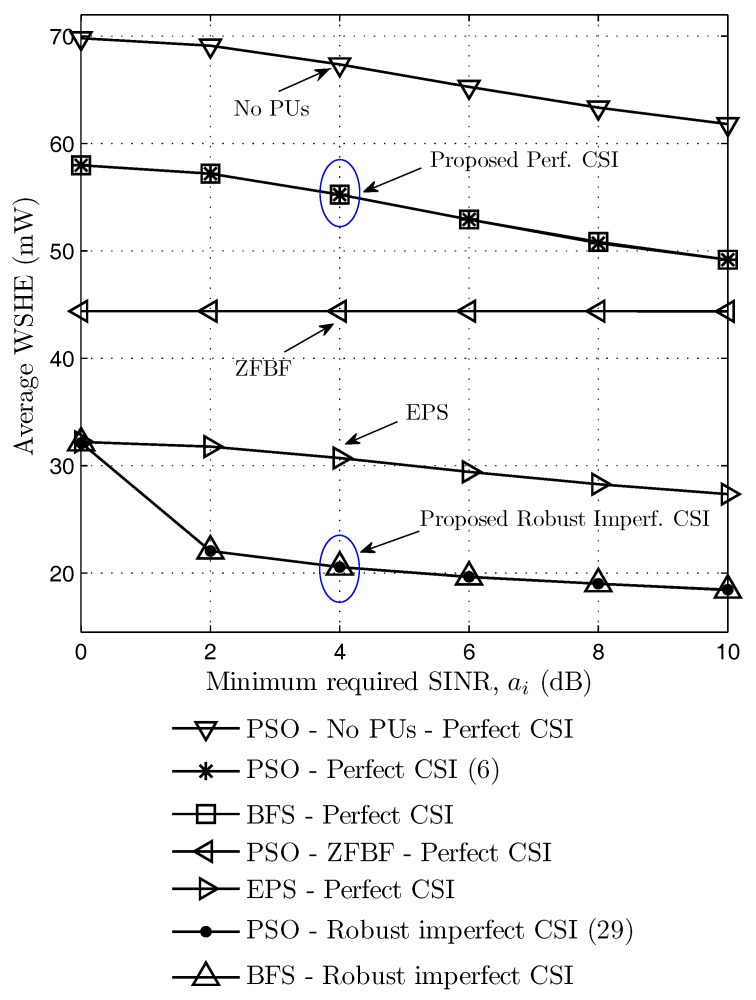
Average weighted sum harvested energy according to minimum required SINR. BFS: brute-force search.

Figure 2 and Figure 3 show the convergence characteristics of the WSHE of the proposed scheme according to the number of iterations in both cases of perfect and imperfect CSI, respectively, when the number of antennas, *N*, is 10. We can see that the fitness function (the WSHE) can quickly converge in less than 30 iterations. As a result, we use the maximum iteration number $T_{\max}$ of 30 for futher simulations. Furthermore, it is observed that the increase of the minimum required SINR corresponds to the decreases of the WSHE of which reasons will be explained in Figure 4 in more detail.

Figure 4 shows average weighted sum harvested power according to the minimum required SINR when the interference threshold $I_{t}=-45\;\mathrm{dBm}$, and the number of antennas N=10. Two suboptimal zero-forcing beamforming (ZFBF) and equal power splitting (EPS) ratios are also considered as the baseline schemes in comparison with the optimal design in perfect CSI case. The design parameters of the ZFBF is obtained by the solution of problem (27) in Section 3.3. In case of the EPS design, the SRs equally divides the received signal for the ID part and the EH part, i.e., $\theta_{i}=0.5$, ∀i. Therefore, the optimization problem (28) has only the beamforming vectors variables in the EPS design. Also, the results of problem (6) without the PU constraints are also provided. In addition, the brute-force search (BFS) method with *M*-dimension search for $\theta_{i}$, i=1,...,M, is also considered to check the optimal value obtained by the proposed PSO-based method.

As shown in Figure 4, the maximum weighted sum harvested energy decreases as the required minimum SINR increases in all cases except the ZFBF scheme. The reason is that the secondary receivers should increase the power splitting ratios and adjust the beamforming vectors in order to get more energy in information decoding parts and further to satisfy the increasing SINR constraints. Hence, the secondary receivers harvest less energy in energy harvesting parts. The ZFBF scheme is not sensitive to the SINR value because the SINR constraints in (27c) are easily satisfied with small noise variances. Also, Figure 4 shows that the optimal solution (6) with perfect CSI achieves better WSHE than ZFBF, EPS, and robust imperfect CSI (35) since both power splitting ratios and beamforming vectors are optimized. The case without primary users gives better WSHE since no PU interference constraints are considered. Furthermore, Figure 4 shows that the proposed PSO-based method achieves the same optimal value as BFS method in both perfect and robust imperfect CSI cases.

**Figure 5:**
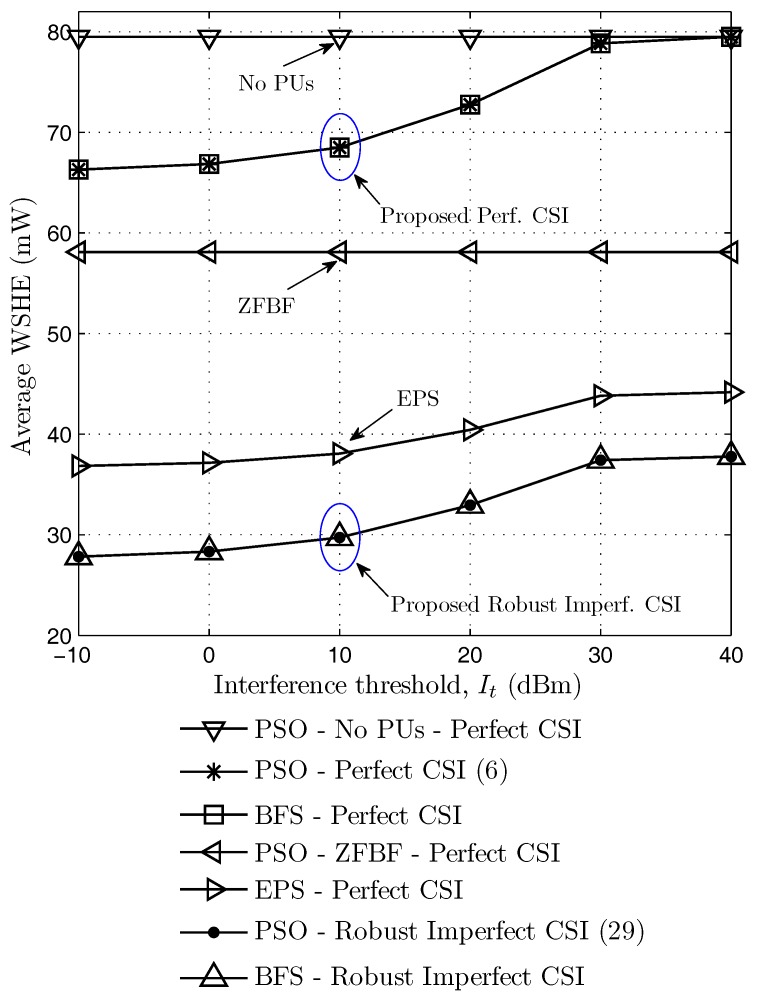
Average weighted sum harvested energy according to the interference threshold.

Figure 5 shows the average weighted sum harvested energy according to the PU interference threshold when the minimum required SINR $a_{i}=5\;\mathrm{dB}$, and the number of antennas N=10. We observe that the WSHE increases with the increasing interference threshold in all schemes except the no PUs and ZFBF schemes. The reason is that when the PU interference threshold increases, the beamforming vectors of the secondary transmitter are more flexible to transmit information and energy simultaneously. The proposed scheme with perfect CSI gives better performance than ZFBF, EPS and the proposed robust schemes. Obviously, the scheme without the constraints of PU interference provides the highest performances. Moreover, when the interference threshold gets smaller, the gap between the proposed perfect CSI and no PUs schemes gets larger because the beamforming vectors have to limit the interference leakage to primary users. On the other hand, when the interference threshold is high, the WSHE of the proposed perfect CSI solution is similar to that of the no PUs scheme because the PU interference constraints are easily obtained. Also, Figure 5 shows the proposed PSO-based method achieves the same performance as the BFS-based method in both perfect and robust imperfect CSI schemes.

**Figure 6:**
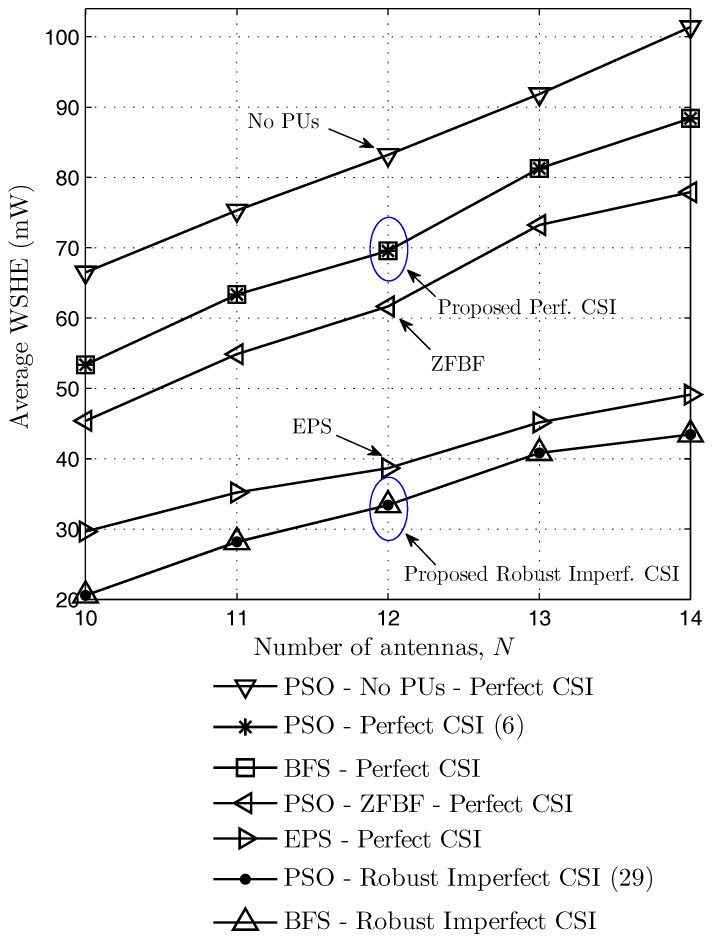
Average weighted sum harvested energy according to number of antennas.

Finally, Figure 6 shows the weighted sum harvested energy according to the number of antennas *N* when the minimum required SINR $a_{i}=5\;\mathrm{dB}$, and the interference threshold $I_{t}=-45\;\mathrm{dBm}$. It is observed that the weighted sum harvested energy increases as the number of antennas increases, in all schemes. When the secondary transmitter has more antennas, it can exploit the extra degrees of freedom in optimizing the direction of the beamforming vectors. Thus, the transmitted signal can be more exactly steered towards the secondary receivers. Consequently, we can obtain more energy harvesting at the secondary receivers and less interference to primary users. Similar to Figure 4 and Figure 5, the performances of the proposed scheme with perfect CSI are better than those of ZFBF, EPS and the proposed robust solution with imperfect CSI. Moreover, the proposed method gives the same harvested energy as that of BFS method in both perfect and imperfect CSI cases while requiring much lower computational complexity.

## 6. Conclusions

We have investigated a multiuser SWIPT cognitive radio network to maximize the weighted sum harvested energy of the secondary receivers under the constraints on total transmit power at the secondary transmitter, the required SINR at the secondary receivers with power-splitting structure, and the interference level at the primary users. The efficient algorithms based on SDR and PSO methods are proposed to find the optimal beamforming at the secondary transmitter and the optimal power-splitting ratios at the secondary receivers for both cases of the perfect and imperfect CSIs. Through simulation, it was shown that the proposed PSO-based algorithm achieves a fast convergence within 30 iterations. It was also shown that the obtained maximum WSHEs in both cases of perfect and imperfect CSI are increased when the allowable interference level at primary users as well as the number of antenna at secondary transmitter get larger.

In the paper, however, the upper bound of WSHE is only archived for the robust imperfect CSI case, and subsequently, the optimal solution satisfying the rank-1 constraints needs to be investigated more in future. In addition, the SDR-based solution has large computational complexity since the number of variables is squared when vector variables are changed to matrix variables. Therefore, the SDR-based approach may not be appropriate to the large-scale system where the antenna number *N* is very large and the complexity of SDR method is $O\left(N^{6.5}\right)$. Thus, fast solutions should be investigated in future works such as with respect to the alternative direction method of multipliers [37].

## Figures and Tables

**Table 1:** Abbreviations and meanings.

Abbre.	Full Form	Meanings
CRNs	Cognitive radio networks [13]	The CRNs use licensed bands of primary networks for communication under overlay or underlay access modes. In this paper, the underlay access mode is applied where the interference from secondary transmitter to primary users is lower than the prescribed threshold.
SWIPT	Simultaneous wireless information and power transfer[1]	In this paper, the transmitter simultaneously sends both information and power to the receivers which are equipped with an information decoder and an energy harvester.
WSHE	Weighted sum harvested energy	WSHE is the sum of all harvested energy of secondary receivers where each value has one weight factor.
CSI	Channel state information	CSI is the complex value of the baseband channel between the transmitter and the receiver.
SDR	Semidefinite relaxation [20]	The fundamental idea of SDR is based on the properties as follows: $X\;=\;xx^{H}\Leftrightarrow X⪰0$ and $\mathrm{rank}\left(X\right)=1$. Thus, we can change the variable from x to X by adding the constraints of X⪰0 and $\mathrm{rank}\left(X\right)=1$. Then, SDR technique removesthe constraint of $\mathrm{rank}\left(X\right)=1$ to obtain the semidefinite programming problem solved by the numerical solver CVX [21]. This optimal solution needs to to be checked for satisfying the rank-1 constraint, or not.
PSO	Particle swarm optimization [22]	PSO is a heuristic evolutional search algorithm which is based on simulating a swarm of particles (birds, fish …) which share information of positions and fitness values with each other. For each iteration, PSO basically performs three steps, called velocity update, position update, and evaluation of the fitness function. Both deterministic and probabilistic rules are used to search overall design space. Each particle moves to a new position with likely improvement of fitness value.
ZFBF	Zero-forcing beamforming	In the ZFBF case, the transmitter designs the beamforming vector, bringing the intended message for one user and being orthogonal to the CSI of other users. Thus, ZFBF design does not cause interference to other users.
EPS	Equal power splitting	The receivers will share half the power of theincoming signal for information decoder, and half for the energy harvester.

**Table 2:** The PSO-based algorithm for solving the WSHE problem (6).

1:	input parameters: $T_{\max}$, $N_{S}$, $\theta_{\min}$, $v_{\max}$, $i_{w}$, $c_{1}$, $c_{2}$,
	and variables $\left\{x_{n}\right\}$, ∀n.
2:	**initialization:**
2:	Assign the iteration index of PSO loop: m=1.
3:	The $x_{n}$’s values of elements, ∀n, are
	assigned randomly in $\left[\theta_{\min},1\right]$, then obtain $f\left(x_{n}\right)$ by solving (8).
4:	Assign the global maximum value: $f\left(g_{b}\right)=\max_{1\leq n\leq N_{S}\;}\;f\left(x_{n}\right)$.
5:	Set the best position of particle: $p_{b,n}=x_{n},\forall n$.
	Set the velocity of particle: $v_{n}=0,\forall n$.
6:	**repeat***(PSO loop)*
7:	**for** $n=1:N_{S}$ **do**
8:	Calculate particle’s new velocity:
	$v_{n}\leftarrow i_{w}v_{n}+c_{1}\pi_{1,n}⊙\left(p_{b,n}-x_{n}\right)+c_{2}\pi_{2,n}⊙\left(g_{b}-x_{n}\right)$,
	⊙ denotes the Hadamard product, and the vectors $\pi_{1,n}$, $\pi_{2,n}$
	have independent uniformly distributed elements in $\left[0,1\right]$.
9:	Restrict vector $v_{n}$’s each element in $\left[-v_{\max},v_{\max}\right]$.
10:	Calculate particle’s new position: $x_{n}\leftarrow x_{n}+v_{n}$.
11:	Restrict each element of vector $x_{n}$ in $\left[\theta_{\min},1\right]$.
12:	Accessment: Compute $f\left(x_{n}\right)$ and the optimal beamforming
	variables ${\left\{w_{i}\right\}}_{n}$, ∀i from the solution of problem (8)-SDR
	according to the PS ratios set, $x_{n}$.
13:	Update the new best position of particle:
	**if** $f\left(x_{n}\right)>f\left(p_{b,n}\right)$ **then**
	Assign: $p_{b,n}\leftarrow x_{n}$.
	**end if**
14:	Update particle’s new global best position:
	**if** $f\left(x_{n}\right)>f\left(g_{b}\right)$ **then**
	Assign: $g_{b}\leftarrow x_{n}$, $\left\{w_{i}^{*}\right\}\leftarrow {\left\{w_{i}\right\}}_{n}$, ∀i.
	**end if**
15:	**end for**
16:	Update iteration index: m←m+1.
17:	**until** $m>T_{\max}$ *(end PSO loop)*
18:	**final results:** the global best value $f\left(g_{b}\right)$ is the optimal value of
	WSHE problem (6) according to the optimal PS ratios $\left\{\theta_{i}^{*}\right\}=g_{b}$,
	and the optimal beamforming vectors $\left\{w_{i}^{*}\right\}$, ∀i.
